# A Systematic Review on Visualizations for Self-Generated Health Data for Daily Activities

**DOI:** 10.3390/ijerph191811166

**Published:** 2022-09-06

**Authors:** Sung-Hee Kim

**Affiliations:** Department of Industrial ICT Engineering, Dong-Eui Univesrity, Busan 47340, Korea; sh.kim@deu.ac.kr

**Keywords:** self-generated health data, data visualization, visualization literacy, consumer health information

## Abstract

Due to the development of sensing technology people can easily track their health in various ways, and the interest in personal healthcare data is increasing. Individuals are interested in controlling their wellness, which requires self-awareness and an understanding of various health conditions. Self-generated health data are easily accessed through mobile devices, and data visualization is commonly used in applications. A systematic literature review was conducted to better understand the role of visualizations and learn how to develop effective ones. Thirteen papers were analyzed for types of data, characteristics of visualizations, and effectiveness for healthcare management. The papers were selected because they represented research on personal health data and visualization in a non-clinical setting, and included health data tracked in everyday life. This paper suggests six levels for categorizing the efficacy of visualizations that take into account cognitive and physical changes in users. Recommendations for future work on conducting evaluations are also identified. This work provides a foundation for personal healthcare data as more applications are developed for mobile and wearable devices.

## 1. Introduction

There are initiatives to return personal health data to patients because they can benefit from increased comprehension of their health status, engagement in care with caregivers, and motivation to choose positive health behaviors [1]. Personal health data allows individuals to become more proactive participants in their health and well-being [2]. Nonetheless, individuals are increasingly capable of generating and collecting their health data outside the clinical setting using mobile technologies. Due to the massive development of sensing technology, several applications support personal health management through smartphones or wearable devices. The data types collected during daily life can also be diverse; they include step counts from activity trackers, nutrition consumption, and sleep cycle data. These data are called self-generated health data [3]. Some data such as blood pressure and blood sugar level can be collected personally, however, may need professional medical knowledge to interpret. Along with this data, health history, symptoms, and treatment history data are called patient-generated data [4]. We distinguish the two and focus on self-generated data as they can be more generally used and understood by the general public.

The interest in personal data collection within the healthcare domain has widely increased. However, we are still in the early stage of designing effective methods of representing self-generated health data and fulfilling the diverse needs of users. Studying how people understand and interpret these self-generated results is important. Visualizations are commonly used to help individuals interpret and contextualize their health data [5]. Data visualizations are known to convey data intuitively by leveraging humans’ visual ability to perceive differences in sizes, colors, and spatial positions [6]. Although research exists on ways to make visualizations efficient and effective, research on self-generated health data is limited. In this study, we investigated how visualizations are used; what kind of data they use, how they impact users, and which factors should be considered for proper visualization design. Users can easily interpret and understand data that reflect their daily activity and lifestyle patterns. We conducted a systematic literature review on related studies of visualizations for self-generated data in the healthcare domain. We only included data collected in our daily life, which does not need medical knowledge to interpret.

When designing visualizations, it is important to define the actions that should be made by the users, determine what task should be supported, and whether the data and visualization were properly selected. To understand the various types of visualizations and how they impact the users, we analyze visualizations and different levels of efficacy such as physical and cognitive impacts. We hope our analysis of visualizations, different levels of efficacy of the applications, and understanding of individual differences will help further research on developing effective visualizations.

## 2. Related Works

### 2.1. Self-Tracking Data

Due to the pervasive ownership of mobile devices, extensive data-capture enables individuals to track their health data manually and passively. For example, 7 out of 10 United States adults track a health indicator (e.g., blood pressure, weight, food consumption, blood sugar, sleep) [7,8]. These self-generated data can be categorized in different ways. First is data collected for medical purposes with professional guidance, such as blood test results [9]. Users mostly collect these data in clinical settings to manage certain diseases like diabetes. Another type of data is collected automatically by tracking devices; examples include step counts [10] and sleep waves [11]. These data are used not only for patients but also by individuals who are interested in their health in daily activities. Lastly, some applications collect self-reported data such as personal mood through questionnaires [12]. These are mostly used for treating mental illness and rely on data that patients enter daily.

To understand the role and effectiveness of using personally generated health data, several systematic review studies have been conducted. The studies can largely be classified by studies in a clinical setting and in personal daily life. Eventually, the difference comes from how the patients could improve their health based on the data. First, in clinical practice, 21 studies showed that decision support was the main role for clinicians [13]. The authors were positive that data from patients will be part of the health care system, however, we need further study on understanding its impact on health outcomes, costs, and patient satisfaction. Additionally, Dinh-Le et al. conducted research on 10 start-up organizations that have developed wearable technology and enabled EHR integration for health systems [14]. The companies were still addressing the challenges of the meaningful use of device data. However, the majority of the companies acknowledged that technology enhances communications, self-constraint, and potential solutions to mitigate existing challenges.

On the other hand, there are also review studies on the use of personal health data in daily life. The first study focused on utilizing technology in diabetes self-management education and support services [15]. A total of 25 studies showed that technology supported healthy eating, being active and metabolic monitoring, and improvement in communication. At last, 13 studies were analyzed to explore the influence of mobile health applications on various dimensions of patient and healthcare provider relationships. The results showed that the use of mobile health applications influenced communication and relationship between the healthcare service providers and the patients [16].

### 2.2. Visualization

Well-designed visualizations have been shown to help fill the gap between low and high health literacy [17]. Visualizations include graphs, diagrams, and infographics, all of which present data with visual components (e.g., shape, color, and size). The rationale behind visualizations is that they attempt to leverage users’ visual skills while reducing cognitive demand for numeracy competencies.

To better understand visualizations, we categorized them into data visualization and infographics. Data visualization or information visualization is “the use of computer-supported, interactive, visual representations of abstract data to amplify cognition” [6]. These representations include simple graphs such as bar charts and new types of visualizations such as treemaps [18], which are not taught in the U.S. K-12 education system. Infographics often contain graphical elements drawn for a particular for a certain scenario or narrative. Data visualizations can be automatically generated even when the data changes, while infographics usually need manual graphical work for each case [19]. And as you see from the definition, interaction techniques such as filtering or zooming are essential for data visualizations.

Data visualization has played an important role in helping understand data, enhancing communication [20], or help the decision-making process to make better decisions [21,22]. Visualizations are also known to lower the cognitive load [23] which can allow users to glance through the data, but still getting a proper understanding while using mobile applications. Most self-tracking devices and their companion mobile apps provide different forms of data representation. However, due to the limited mobile display size, simple and static visualizations are commonly used (e.g., bar graphs in the FitBit app). There have several studies on evaluating the visualization on desktop environments [24,25]. To measure the effectiveness properly, selection of visualizations, task to be measured, expertise on the domain data and visualization, and measures for effectiveness should be carefully designed.

## 3. Methods

**Figure 1:**
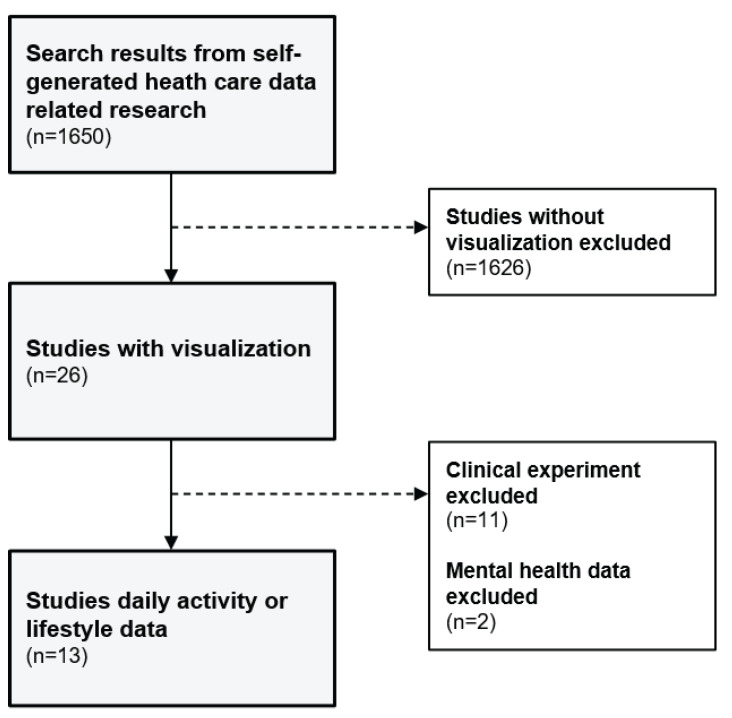
Stages of the search process and number of selected studies at each stage.

In June 2022, we searched scholarly databases such as PubMed, IEEE Xplore, ACM Digital Library, Scopus, and Google Scholar for articles pertaining to self-generated health data and visualizations. We selected these databases to capture various fields, including medicine, medical informatics, and computer science. Our search terms included a combination of terms such as self-generated health data, patient-generated health data, health data, data visualization, graph, daily activity, physical activity, daily habits, daily health, steps, sleep, and diet. We included studies written in English, studies of self-generated health data with applications of data visualization, and qualitative or quantitative experiments. We excluded studies completed in a clinical setting because we wanted to focus only on data that can be easily understood without medical knowledge. Domain knowledge is known to be important when interpreting visualizations and we wanted to minimize the effect of this, therefore, selected applications that utilize personal data that can be easily collected in our daily life. Additionally, we excluded research on mental health because most studies on mental health used survey questionnaires to collect data. The author also screened papers while reading titles and abstracts against the eligibility criteria. The screening process is shown in Figure 1. Initially, we searched 1650 papers, and from the screening criteria, we eventually were left with 13 papers. For analysis, as the number of papers were only 13, a qualitative approach was applied to analyze the types of visualizations, the effectiveness of visualizations, and different level of efficacy for physical and cognitive levels.

## 4. Results

In this section, we report the results of our analysis of 13 papers. First, we report the data that these studies collected and the visualization characteristics that they presented. This provides an overview of how visualization is utilized for personal health data within daily activities. We also analyze how the visualization was useful for the users. Based on the studies’ results regarding the efficacy of visualizations, we then suggest different levels of efficacy for users.

### 4.1. Visualization Characteristics

**Figure 2:**
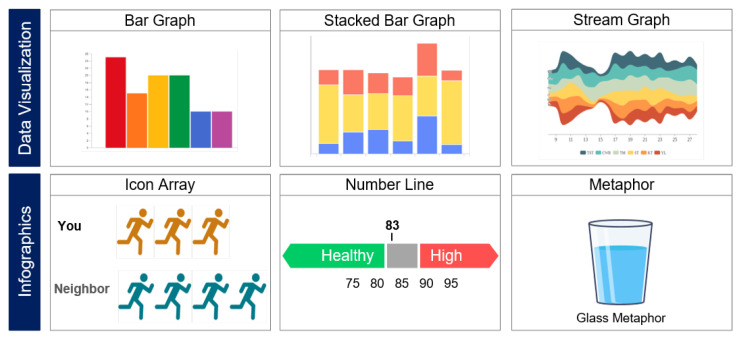
Stages of the search process and number of selected studies at each stage.

After reviewing 13 visualization studies about self-generated health data for daily activities, we determined that most of the studies related to lifestyle habits such as activity routines, as reflected by step counts, sleep cycles, and diet. We are largely able to divide visualizations into two categories: data visualization and infographics by representation. Data visualization was used to faithfully represent the collected data without visual decoration. When data included icons and metaphors, we classified them as infographics. Figure 2 includes examples of data visualizations and infographics that are similar to what has been reported in the literature. We categorized visualizations according to our definitions even when study authors specified their research topic as infographics. Data visualizations explicitly represent the numeric data in visual encoding such as height or length. On the other hand, infographics mostly have decorations using icons, additional visual aspects, or metaphors.

Table 1 reports types of data, representation type (i.e., data visualization and infographics), visualization type, and the presence of interaction techniques in the visualization for each paper. For the types of data, there were data that is collected through sensor devices such as step count, heart date, active calories, Body Mass Index (BMI), and sleep cycles. Other types of data needs to be entered by the user such as nutrition consumption, physical activities, or weight.

After analyzing the frequency of visualization representation use, we determined that six studies used only data visualization among 13 studies. Two studies only used infographics, and five used data visualization and infographics together. Of the 11 studies using data visualization, bar graphs were the most considered visualization type with nine cases. Four studies considered line graphs, and three considered stacked bar charts. Bar graphs are known to provide comparison easily and line graphs provide trend information to the users. As most of the data is collected continuously, timeline or calendar-based visualizations were also used to support the time series characteristics. Only two studies considered the user’s dynamic interaction with visualization results, such as touching and swiping. One of the studies was to evaluate commercial services that implemented a high level of interaction techniques [11,26]. Only one study conducted a specific experiment on the impact of visual aspects. Eikey et al. manipulated the color of an exercise progress bar and examined how the presentation of feedback impacts the users [27].

Among the studies about infographics, in the order of number line types, icon arrays, and metaphors of commonly known figures were used. Number line visualization is a visual representation that includes numerical information in an ordered manner. Based on the data, a tick shows the user’s position, and indicates whether the value is normal, healthy, or in a dangerous section. Icon array is a representation with one shape repeatedly which can be easily interpreted for counts. Several metaphors were used to help the users intuitively understand the data such as cloverleaf petals and flowers in the garden to show progress and achievement level, or glass metaphors for water consumption.

Regarding individual characteristics, two studies considered different levels of health literacy of users and developed personalized visualizations [28,29]. For different age groups, two studies investigated older adults as they could have different digital literacy levels and experiences with these types of digital devices [26,30].

**Table 1:** Characteristics of visualizations including types of data presented, visualization type, and the presence of interaction technique.

First Author (Year)	Types of Data	Visualization Characteristics		
		Representation	Visualization Type	Interaction
Alrehiely et al., 2018 [15]	Step count, heart rate, active calories	Data visualization	Bar graph Line graph	Static
		Infographics	Flowers and garden metaphor Clock and calendar metaphors	Static
Arcia et al., 2013 [28]	Vegetable servings per week, exercise per week, Body Mass Index (BMI), waist circumference, sleep, energy, nutrition, physical activity	Data visualization	Bar graph Adapted hGraph ^1^	static
		Infographics	Reference category silhouettes ^2^ Icon array Control panel analogy	static
Arcia et al., 2015 [31]	Body Mass Index (BMI), physical activity, overall health, fruit & vegetable consumption	Infographics	Number line Cloverleaf petal	static
Arcia et al., 2016 [29]	Exercise, sleep, diet	Infographics	Number line Icon array	Static
Beaudin et al., 2006 [32]	Weight, step counts, nutrition, water consumption, daily routines	Data visualization	Dot graph Stacked bar graph	Static
		Infographics	Body map Glass metaphor	Static
Brewer et al., 2012 [27]	Body Mass Index (BMI)	Data visualization	Bar graph	Static
Choi et al., 2018 [11]	Sleep data	Data visualization	Bar graph Line graph Stacked bar graph Stream graph	Interactive
Ehn et al., 2018 [26]	Step count, sleep cycles	Data visualization	Bar graph Line graph	Interactive
Eikey et al., 2015 [27]	Step count	Data visualization	Bar graph	Static
Frackleton, 2021 [30]	Step count, walking distance, calories burned	Data visualization	Bar graph Calendar type bubble plot Calendar type heat map Stacked bar graph	Static
Maškanceva, 2020 [33]	Sleep data, coffee consumption, exercise	Data visualization	Bar graph Timeline based bar graph	Static
		Infographics	Clock-shaped visualization	Static
Meyer et al., 2016 [34]	Step count, calories burnt, sleep cycle, and weight	Data visualization	Bar graph Line graph Ring and bubble graphs	Static
Schneider et al., 2017 [35]	Water consumption	Data visualization	Donut chart	Static
		Infographics	Creature metaphor Glass metaphor	Static

^1^ Series of gender-specific body silhouettes corresponding to body mass index /waist circumference reference categories. ^2^ An adaptation of the “hGraph” [36]. Individual variables are plotted radially around a donut figure in which the figure represents optimal reference ranges.

### 4.2. Level of Efficacy

In this section, we analyzed the efficacy reported in the self-generated health data visualization studies. We found that there are different levels of utility and can be largely divided into physical impact and cognitive impact. Physical impact emphasizes the practical change of human behavior and cognitive impact emphasizes the effect on the human mind. A total of six efficacy levels were defined; four levels for cognitive impact and two levels for physical impact. In Table 2, the bottom four rows refer to low-level impact and the top two rows reflect higher-level impacts.

This section defines each efficacy level and includes exemplar quotes or actions from related studies. First, the cognitive side has four levels: attract interest, enhance self-awareness, enhance motivation and self-efficacy, and gain insight. Attract interest refers to arousing curiosity, creating fun for users, and encouraging them to pay attention to their health conditions. In one stud of home monitoring systems that provided data on movements, participants cited the display as “providing entertainment” [32]. Enhance self-awareness refers to providing information so users can understand their health status and acquire relevant knowledge. Alrehiely et al. evaluated different types of visualizations of step count, heart rate, and active calories. A majority of the participants in their study were university students who stated that the visualizations were effective in “data comprehension and gaining knowledge (on personal data)” [10]. Ehn et al. investigated how seniors experience using activity monitors for physical activities in daily life. After using commercial apps with tablets for 9 days, one participant said, “Yes, I was positively surprised that I had taken so many steps. I hadn’t walked that much (laugh). I was positively surprised” [26]. *Enhance motivation and self-efficacy* refers to visualizations that encourage users to take appropriate actions based on visualized information about their health status. Schneider et al. conducted a study on promoting water consumption with visualizations and reported the following motivating effect, “I feel motivated to drink more water after looking at the visualization” [35]. Lastly, *gain insight* refers to visualizations providing users with meaningful interpretations and new perspectives, such as gaining a perspective on their lifestyle and reevaluating their life goals. The following statement describes this well, “Having capacity to visualize sleep data in clear and informative graphs could further help users easily understand their sleep patterns and facilitate meaningful interpretations of the data” [11].

For the physical side, *promote behavioral change* refers to visualizing personal health data that motivates users to increase or maintain activity. In one study, a participant using activity monitors mentioned that the visualization of physical activities helped them increase their activity: “I get pushed if I have been too lazy.” [26]. *Promote effective self-care and community health* refers to inducing effective self-management of individual behavioral changes and further promoting the community’s health. Arcial et al. used tailored infographics to promote community health and support community members’ comprehension of their health information [31]. The goal of these personal visualizations of health data is to help users understand their health status, provide feedback, and learn to be proactive in their health management by changing behaviors.

Among the various levels of utility, the most cited utility was “increasing an individual’s understanding of health status.” This is often individuals’ first step in engaging with their data, and it can eventually lead them to achieve higher levels of utility in visualizations. The next reported effect was “change in individual’s behavior” shown in the visualizations as a feedback mechanism. A majority of the research emphasized the positive effect of visualizations on users, however, Schneider et al. mentioned the negative effects of a poorly designed visualization, which can deter individuals from engaging with visualizations. This could come from a bad selection of the visualization, preference for certain visualizations, or personal differences in the ability to understand data or comprehend visualizations. Related work on data literacy and data visualization literacy is also becoming an important topic, especially as these abilities are critical when using visual interfaces [37].

**Table 2:** Different levels of the efficacy of visualizations representing self-generated health data.

Category	Efficacy	Definition	Related Papers
Physical	Promote effective self-care and community health	Inducing effective self-management of individual behavioral changes and further promoting community’s health	[31]
	Promote behavioral change	Visualize of personal health data that motivates users to increase or maintain activity	[11,26,29,32,33]
Cognitive	Gain insight	Providing users with meaningful interpretations and new perspectives, such as gaining a perspective on their lifestyle and reevaluating their life goals	[11,32]
	Enhance motivation and self-efficacy	Encourage users to take appropriate actions based on visualized information about their health status	[10,26,27,35]
	Enhance self-awareness	Provide information so users can understand their health status and acquire relevant knowledge	[10,11,26,28,29,30,31,33,34,38,39]
	Attract interest	Arousing curiosity, creating fun for users, and encouraging them to pay attention to their health condition	[10,29,32,35]

### 4.3. Impact of Individual Differences on Visualizations’ Effectiveness

Health data visualization can improve individuals’ understanding and level of health literacy, and research is being conducted to develop more intuitive and effective visualization [28,33]. However, the degree to which an individual understands visualized health data likely varies according to their health literacy, data literacy, digital literacy, and visualization literacy. In other words, an individual’s level of understanding of visualization may vary depending on their visualization literacy [37]. Data visualization literacy is the ability of a person to read, interpret, and construct data visualizations. To make sure that visualization is properly used or evaluated by users, service providers or researchers should take this factor into consideration and understand the level of literacy of their main audience. Researchers may need to administer visualization literacy tests to patients or users to properly understand their behavior.

## 5. Discussion

To increase the effectiveness of visualizations in self-generated health data, we suggest researchers and service providers consider the following key points. First, visualizations should be carefully designed by the selection of data to be shown, what type of visualization is effective, and what task should be supported by the visualization. We believe that Table 1 and Table 2 can guide the designers or developers to set the goal of the visualization and design accordingly understanding the variety of choices. Especially, as shown in Table 2, the providers should define on what level the visualization and the app should support the user, and design the task that should be fulfilled with the visualization.

Additional factors also should be taken into consideration. As users will have varying visual literacy skills, researchers or service providers need to educate users so that they can comprehend data properly, and they should measure users’ visualization literacy level before introducing them to visualizations. Specific action guides for each visualization could be provided. Second, proper visualizations should be selected according to the data type and the main task that the developers indicated. Different visualizations support different tasks; bar charts are good for reading single data points and comparing items, while line charts help to read trends and changes over time [40]. Most data collected from tracking devices are time series data. However, several applications lack providing line charts or the ability to manipulate the time axis of visualization results. Visualizing data over time is also an important factor for comparing and providing feedback for behavioral changes shown in the data. Additionally, interactive visualizations are also important to help users engage with their data. Filtering dates or zooming actions with the finger can help users easily explore time series data.

We need a systematic way to evaluate visualizations to test their effectiveness and see whether certain visual interfaces can elicit different cognitive or physical changes. Currently, there are no consistent approaches for evaluating visualizations in the healthcare domain. Several factors can differ based on the type of data and the goal pursued, such as visualization selection, how to measure visualizations’ effectiveness, how long the study should take, personal literacy levels, and the need for education on the data or visualization. Even for a single visualization design, the selection of color, layouts, narrative texts, interactive elements, metaphor use, and number of visualizations shown in the display can all affect the readability or perceived usefulness to the user. Therefore, careful curation of these aspects is needed for proper experiments. If researchers conduct studies consistently and report them in similar formats, the results can be accumulated and eventually help to systematically approach different scenario usages.

For further research, we need to investigate the effectiveness of data visualizations compared to infographics. Even with the same data, different representations could affect curiosity, familiarity, and engagement levels. For data visualizations, additional research can be done on the effectiveness of visualizations related to certain data types in the mobile environment, which visualizations have a better impact on the behavioral changes, and the impact of longitudinal studies. For infographics, we can investigate the impact of artistic factors, the impact of narrative texts, and how users perceive metaphors in visualizations. When we have enough research in this field, we can come up with a theoretical framework, checklist, or design guidelines on visualization design for self-generated health data so that one can ensure that visualization would fulfill users’ needs and help them meet their goal.

## 6. Conclusions

Through a systematic literature review, we can understand that data visualizations can have a valuable impact on self-health management, empowering individuals to make cognitive and physical changes. To design better visualizations, we need to understand the characteristics of data visualizations, how these visualizations can affect users, and the individual differences among users. More attention is needed to develop and evaluate visualizations of certain data types. We find opportunities for more robust data collection and reporting and more systematic methods for evaluation that will help provide guidelines in this domain. Personal health data in nonclinical and clinical settings will continuously increase, and new digital health tools will undoubtedly be developed. We hope that this research will provide a foundation for future research.

Through this study we have analyzed the factors focused on the types of visualizations and the level of efficacy. However, to derive specific guidelines, we need to analyze specific task types, duration of the use of the service, integration level with clinical practice, and whether the applications are for only personal health or chronical management. Visualizations are sensitive and have the freedom of several design spaces. We believe that studies on several purposes will lead to effective visual interfaces.

## Data Availability

Not applicable.
